# Fern spore extracts can damage DNA

**DOI:** 10.1054/bjoc.2000.1204

**Published:** 2000-06-02

**Authors:** S E Simán, A C Povey, T H Ward, G P Margison, E Sheffield

**Affiliations:** CRC Carcinogenesis Group, CRC Drug Development Group, Paterson Institute for Cancer Research, Christie Hospital, Manchester, M20 4BX; School of Biological Sciences, 3.614 Stopford Building, The University of Manchester, Oxford Road, Manchester, M13 9PT; School of Epidemiology, Medical School, The University of Manchester, Oxford Road, Manchester, M13 9PT, UK

**Keywords:** fern spores, DNA strand breaks, single-cell gel electrophoresis (comet) assay

## Abstract

The carcinogenicity of the vegetative tissues of bracken fern (*Pteridium*) has long been established. More recently, the carcinogenic effects of the spores of bracken have also been recognized. Both vegetative tissues and spores of bracken can induce adducts in DNA in animal tissues, but the possible genotoxic or carcinogenic effects of spores from fern species other than bracken are unknown. The single-cell gel electrophoresis (‘comet’) assay was used to investigate whether fern spores can cause DNA damage in vitro. Extracts of spores from six fern species were administered to cultured human premyeloid leukaemia (K562) cells. Spore extracts of five fern species: *Anemia phyllitidis*, *Dicksonia antarctica*, *Pteridium aquilinum*, *Pteris vittata* and *Sadleria pallida*, induced significantly more DNA strand breaks than those in the control groups. Only in one species, *Osmunda regalis*, was the effect no different from that in the control groups. Using extracts from *A. phyllitidis* and *P. vittata*, the extent of DNA damage was increased by increasing the original dose 10 times, whereas an experiment in which exposure times were varied suggested that the highest levels of strand breaks appear after 2 h exposure. Simultaneous incubation with human S9 liver enzyme mix ablated the damaging effect of the extracts. Our data show that fern spore extracts can cause DNA damage in human cells in vitro. Considering the strong correlation between DNA damage and carcinogenic events, the observations made in this report may well have some implications for human health. © 2000 Cancer Research Campaign


British Journal of Cancer (2000) **83**(1), 69–73
© 2000 Cancer Research Campaign
doi: 10.1054/ bjoc.2000.1204, available online at http://www.idealibrary.com on IDEAL®

### Fern spore extracts can damage DNA

**SE Simán[1,3], AC Povey[1,4], TH Ward[2], GP Margison[1] and E Sheffield[3]**

[1]CRC Carcinogenesis Group, Paterson Institute for Cancer Research, Christie Hospital, Manchester, M20 4BX; [2]CRC Drug Development Group, Paterson Institute for Cancer Research, Christie Hospital, Manchester, M20 4BX; [3]School of Biological Sciences, 3.614 Stopford Building, The University of Manchester, Oxford Road, Manchester, M13 9PT; [4]School of Epidemiology, Medical School, The University of Manchester, Oxford Road, Manchester, M13 9PT, UK

**Summary** The carcinogenicity of the vegetative tissues of bracken fern (*Pteridium*) has long been established. More recently, the carcinogenic effects of the spores of bracken have also been recognized. Both vegetative tissues and spores of bracken can induce adducts in DNA in animal tissues, but the possible genotoxic or carcinogenic effects of spores from fern species other than bracken are unknown. The single-cell gel electrophoresis ('comet') assay was used to investigate whether fern spores can cause DNA damage in vitro. Extracts of spores from six fern species were administered to cultured human premyeloid leukaemia (K562) cells. Spore extracts of five fern species: *Anemia phyllitidis*, *Dicksonia antarctica*, *Pteridium aquilinum*, *Pteris vittata* and *Sadleria pallida*, induced significantly more DNA strand breaks than those in the control groups. Only in one species, *Osmunda regalis*, was the effect no different from that in the control groups. Using extracts from *A. phyllitidis* and *P. vittata*, the extent of DNA damage was increased by increasing the original dose 10 times, whereas an experiment in which exposure times were varied suggested that the highest levels of strand breaks appear after 2 h exposure. Simultaneous incubation with human S9 liver enzyme mix ablated the damaging effect of the extracts. Our data show that fern spore extracts can cause DNA damage in human cells in vitro. Considering the strong correlation between DNA damage and carcinogenic events, the observations made in this report may well have some implications for human health. © 2000 Cancer Research Campaign

**Keywords:** fern spores; DNA strand breaks; single-cell gel electrophoresis (comet) assay

Several plants contain mutagenic substances (Basaran et al, 1996), but it is reported that only bracken fern (*Pteridium*) causes cancer naturally in animals (Prakash et al, 1996). In 1960, Rosenberger reported a correlation between the long-term intake of *Pteridium aquilinum* and a cattle disease called bovine enzootic haematuria, which is characterized by chronic haemorrhage from neoplasias in the urinary bladder mucosa (Pamukcu, 1963). Evans and Mason (1965) found that rats fed dried bracken fronds (leaves) developed gastrointestinal tumours and guinea pigs fed experimentally with dried young shoots of *Pteridium* developed urinary bladder tumours (Bringuier et al, 1995). Even milk from cows feeding on a bracken-containing diet has been shown to be carcinogenic (Villalobos-Salazar et al, 1990), possibly as a result of the transmission of the bracken carcinogen ptaquiloside from the digestive tract to the milk (Alonso-Amelot et al, 1996).

The carcinogenicity of the spores of bracken has also been recognized (see Simán et al, 1999 for review). Leukaemia, gastric, mammary and lung tumours have been reported in mice, following exposure to *Pteridium* spores (Evans, 1987; Villalobos-Salazar et al, 1995). Both vegetative tissues (Prakash et al, 1996) and spores (Povey et al, 1996) of bracken can induce DNA lesions in the form of adducts in rodent and calf gastrointestinal tissues. Ptaquiloside, generally regarded as the main genotoxin of bracken (Alonso-Amelot et al, 1996), has been shown to bind covalently to DNA-bases (Ojika et al, 1987).

The risk of acquiring cancer from bracken can be decreased by avoiding ingestion of its vegetative tissues, but the spores can be unintentionally breathed in and subsequently ingested. The extent of this problem in relation to other fern species is largely unknown, but in one study performed in Bangkok, Thailand, fern spores were found to be the third most common airborne biological particle in that area, occurring with a frequency of 17%. Only pollen of sedges (23%) and grasses (20%) were more frequent (Bunnag et al, 1989). Human exposure to fern spores is also likely to occur in a range of indoor environments, as many fern species are popular ornamental plants, in public and private gardens, greenhouses and as household pot-plants. When grown under favourable climatic conditions they tend to produce spores throughout the year (Simán and Sheffield, 2000). When sporing, a single bracken frond has been estimated to be capable of producing about 300 million spores (Conway, 1952), and a *Dicksonia antarctica* tree fern frond as many as 750 million spores (Page, 1979). Given that a mature *D. antarctica* tree has about 20 fronds, if the spores are genotoxic, it would be worrying that such plants are used as ornamentals in indoor environments.

The single-cell gel electrophoresis ('comet') assay (Östling and Johanson, 1984) was used to investigate whether fern spores can cause DNA damage in vitro. Extracts of spores from six fern species were administered to cultured human premyeloid leukaemia (K562) cells. The presence of DNA strand breaks in the treated cells was detected by computer analysis of microscopic images.

*Received 21 October 1999*
*Revised 12 February 2000*
*Accepted 15 February 2000*

*Correspondence to:* SE Simán

### MATERIALS AND METHODS

### Extraction of fern spores

Extracts were made from spores of *Anemia phyllitidis* (L.) Swartz, *Dicksonia antarctica* Labill., *Osmunda regalis* L., *Pteridium*

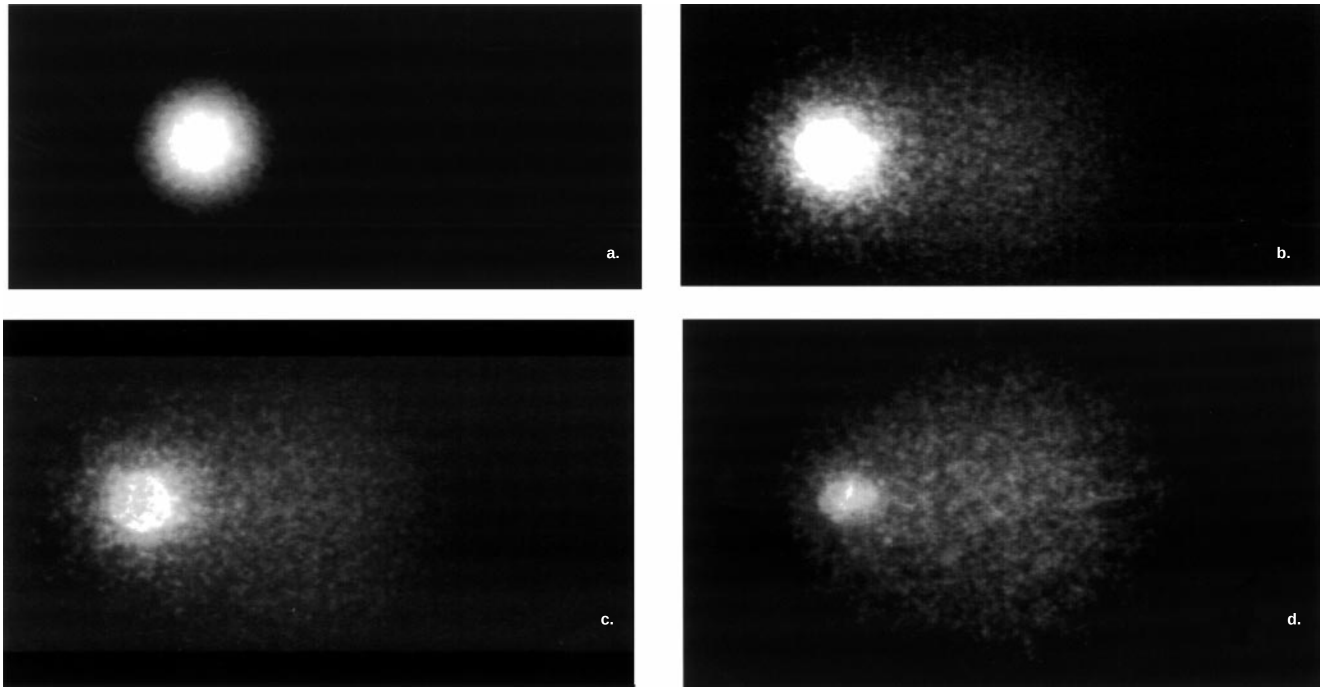

**Figure 1** ‘Comet’ assay images of K562 cells. (**a**) untreated cell, (**b**) cell incubated with *Anemia phyllitidis* spore extract, (**c**) cell incubated with *Dicksonia antarctica* spore extract, (**d**) cell irradiated with 10 Gy $^{137}$Cs

*aquilinum* (L.) Kuhn., *Pteris vittata* Linn. and *Sadleria pallida* Hook. & Arn; fern species chosen to represent a wide morphological, taxonomic and geographic spectrum. Spores, 25 mg of each species, were sonicated in a waterbath for 15 min and then extracted with 2.5 ml DMSO at 37°C overnight. The crude extracts was filtered through a 0.2 µm sterile filter to remove remains of spores and sporangia. The filtered extracts were used immediately after preparation.

In a further set of experiments a series of different spore extract concentrations was prepared, using 12.5–500 mg of spores of *A. phyllitidis* and *P. vittata* extracted in 2.5 ml DMSO as described above.

### Treatment of cells

Human premyeloid leukaemia (K562) cells were incubated with fern spore extracts (2.5 µl extract to ca. $2.5 \times 10^4$ cells), with or without the addition of 10 µl S9 liver enzyme mix (GenTest corporation, UK). Controls were either untreated or incubated with 2.5 µl DMSO, or with 10 µl S9 liver enzyme mix. Cells irradiated with a single dose of 10 Gy ($^{137}$Cs, dose rate 0.4 Gy min$^{-1}$; specific activity 180 Ci) were included with each experiment as a positive control. The amount of DNA damage produced by the extracts was compared to that produced by ionizing radiation. For this purpose K562 cells were irradiated with a range of doses between 0 and 10 Gy, to confirm the linearity of the dose-dependency curve. The cells were exposed to the fern spore extracts for 2 h. In a further study, using *A. phyllitidis* and *P. vittata*, exposure times were 10 min, 30 min, 2 h and 4 h to investigate whether this had any effect on the level of damage caused by the extracts.

### The alkaline single cell gel electrophoresis assay

After treatment the cells were subjected to the alkaline single-cell gel electrophoresis assay as described previously (Ward et al, 1997), but using SYBR® green (Flowgen, UK) instead of propidium iodide. Cells were suspended in 1 ml low-melting-point agarose (1% in PBS) and pipetted onto glass microslides precoated with a thin agar layer (1% agar in $H_2O$). Two slides were prepared for each treatment. The cell-agarose film was immediately covered with a coverslip and transferred to an ice tray to inhibit endogenous DNA repair mechanisms. Once the gels were firmly set, the coverslips were removed and the slides submerged in cold lysis buffer (2.5 M NaCl, 10 mM Tris, 100 mM EDTA, 1% Triton X-100, 1% DMSO, pH 10.5–11.0) for 1 h. The gels were washed three times for 15 min in distilled water, then transferred to a flatbed electrophoresis tank. Alkaline electrophoresis buffer (50 mM NaOH, 1 mM EDTA, pH>12.5) was poured into the tank and the slides were left in the buffer for 30 min. During this stage the DNA strands unwind. Electrophoresis was then performed in the alkaline buffer for 25 min at a voltage of 0.6 V cm$^{-1}$ and a current of 90 mA. The slides were then neutralized by the addition of $2 \times 1$ ml of Tris-HCl pH 8.0 and stained with $2 \times 1$ ml of SYBR®green (0.0001% in DMSO).

### Microscopy and image analysis

The slides were examined at × 250 magnification under an epifluorescent microscope (Zeiss, Germany) illuminated with green light from a 50 W mercury source and using a 580 nm reflector and 590 nm barrier filter set. 25 images from each of the two slides per treatment were captured and analysed using a Sony HAD-1 interline CCD camera and Kinetic Image software (UK). The level of damage was measured through the individual tail moment, which compares the DNA contents of the head and the tail of the comet (Ashby et al, 1995). The mean tail moments from 50 cell images from each of at least four replicates of the different treatments (i.e. 200 per treatment group) were compared statistically using One-way Analysis of Variance.

 © 2000 Cancer Research Campaign

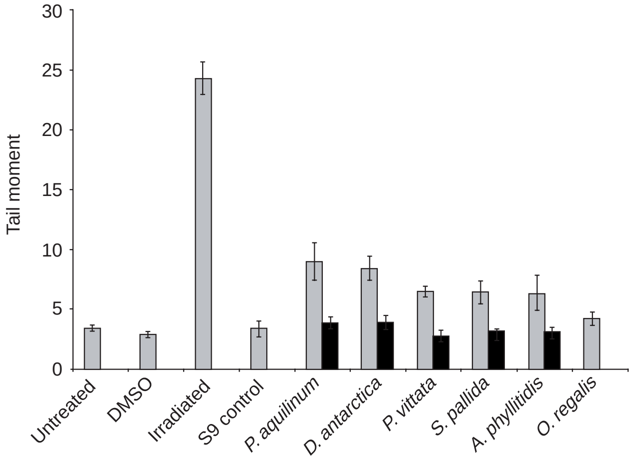

**Figure 2** Tail moment as a measure of the level of DNA damage in human premyeloid leukaemia (K562) cells. Grey bars represent levels of DNA damage in controls and cells incubated with fern spore extracts for 2 h in 37°C. Black bars represent levels of damage in cells incubated with fern spore extracts and S9 liver enzyme mix simultaneously. Spore extracts from five fern species (*P. aquilinum*, *D. antarctica*, *P. vittata*, *S. pallida* and *A. phyllitidis*) induced levels of DNA damage significantly higher than those in the control groups. Error bars show standard error of the mean.

### RESULTS

Spore extracts of *A. phyllitidis*, *D. antarctica*, *P. aquilinum*, *P. vittata* and *S. pallida* induced a significantly higher frequency of DNA strand breaks than found in the various control groups. (ANOVA, LSD, $P < 0.05$, $n \geq 4$: Figures 1 and 2). The most extensive damage was produced by *P. aquilinum* and reached levels three times as high as the background levels in the controls. The comparatively milder effect of *P. vittata* was up to twice as great as the controls. Treatment with spore extract from one species, *O. regalis*, did not show an effect different from that seen in the control groups. Since radiation damage was linearly proportional to dose (data not shown), the highest level of damage produced by the fern spore extracts, at this concentration, would be equal to a dose of 3 Gy. Simultaneous incubation with the S9 liver enzyme mix ablated the damaging effect of the extracts ($P < 0.05$, $n = 4$: Figure 1).

To ascertain whether different levels of DNA damage were caused by different concentrations of extracts, two fern species (*A. phyllitidis* and *P. vittata*) were used. The resulting DNA damage in the cells was significantly higher from the × 10 and × 20 doses than from the × 1 and × 2 doses (Figure 3). However, no significant difference in DNA damage was observed within these pairs, (i.e. × 1 was not different from × 2, and × 10 not different from × 20). The × 0.5 *A. phyllitidis* dose did not result in DNA damage significantly different from that in the controls ($P < 0.1$, $n = 4$). The highest concentrations of spore extracts produced DNA damage at a level corresponding to radiation doses of 5 Gy (*A. phyllitidis*) and 6 Gy (*P. vittata*).

In order to determine the effect of differing exposure times we used spore extracts from *A. phyllitidis* and *P. vittata*. The experiment was carried out according to the same protocol, except that cells were exposed to the spore extracts at the original dose for 10 min, 30 min, 2 h or 4 h. Only after 2 h were the amounts of DNA damage significantly higher than those of the controls (Figure 4). Cells exposed for 4 h showed amounts of strand breaks equivalent to the cells exposed for 10 and 30 min; neither of these three groups was different from the controls ($P < 0.05$, $n = 5$).

### DISCUSSION

The single-cell gel electrophoresis assay is well established as a method for the detection of DNA damage, and corresponds well to other established genotoxicity assays (Wagner et al, 1998; Cebulska-Wasilewska et al, 1998; Leroy et al, 1996). A recent review of the use of this assay shows that 88% of known carcinogens are detectable as such (Anderson et al, 1998). Our data clearly show that fern spore extracts can cause DNA lesions in human cells in vitro. Not only do bracken spores, which are known to be carcinogenic, cause DNA damage, but spores from a wide range of other fern species do too.

The exposure time that gave the highest levels of strand breaks was 2 h. Strand breaks detected by the single-cell gel

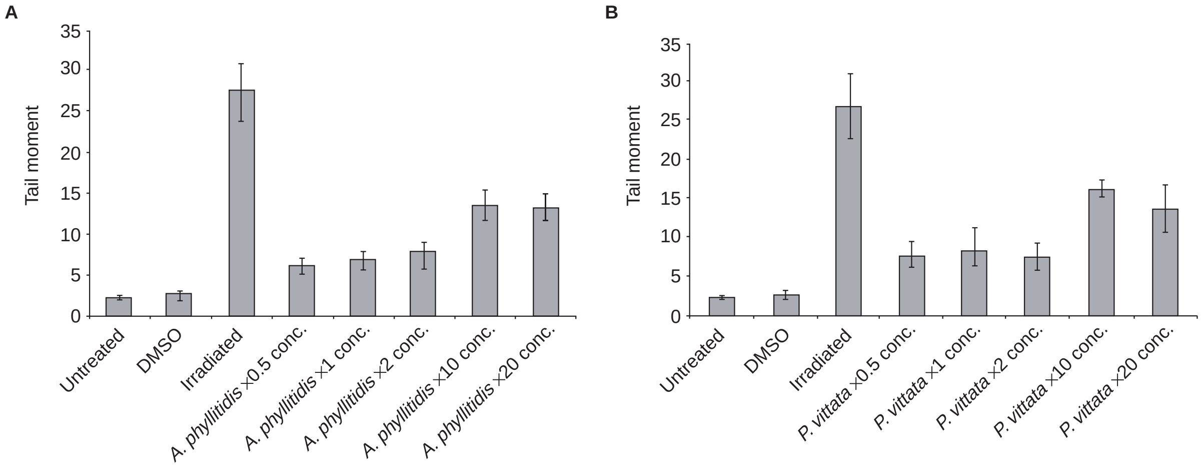

**Figure 3** Tail moment as a measurement of DNA damage in human premyeloid leukaemia cells (K562) incubated with a series of different concentrations of fern spore extracts. The extract concentration used in the original experiment (25 mg spores to 2.5 ml DMSO) is set to × 1. A tenfold increase of this concentration resulted in significantly increased levels of DNA damage both in cells incubated with spore extracts of *Anemia phyllitidis* (**A**), and those incubated with spore extracts of *Pteris vittata* (**B**). Error bars show standard error of the mean.

*© 2000 Cancer Research Campaign*

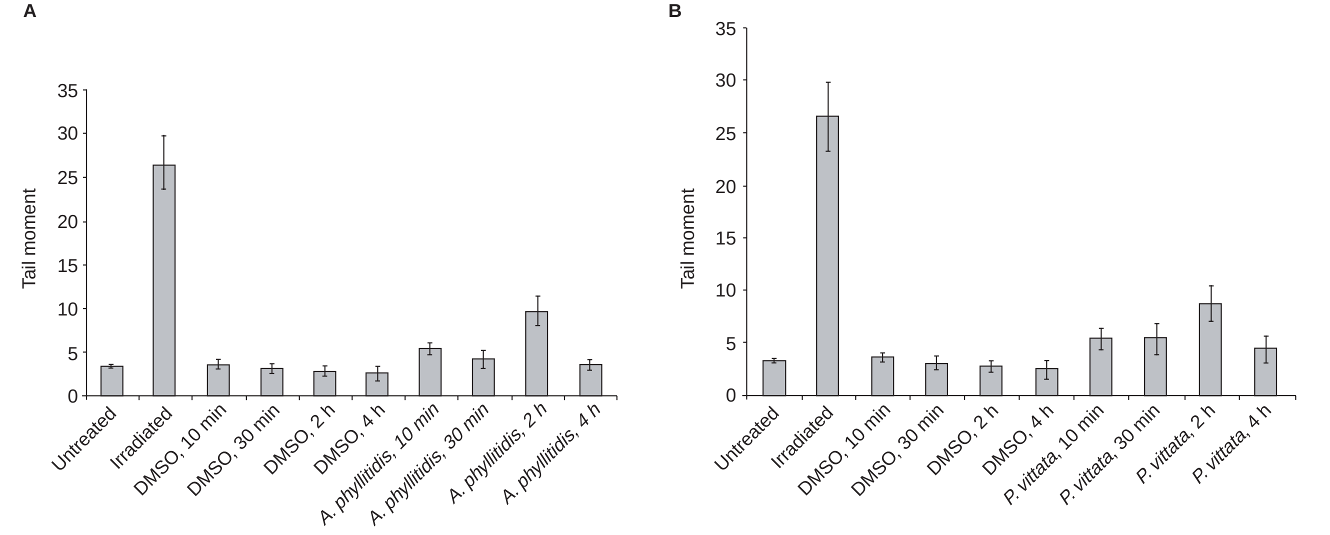

**Figure 4** Tail moment as a measurement of DNA damage in human premyeloid leukaemia cells (K562) incubated with spore extracts for 10 min, 30 min, 2 h and 4 h. The levels of DNA damage were highest after 2 h both in cells incubated with spore extracts of *Anemia phyllitidis* (**A**), and those incubated with spore extracts of *Pteris vittata* (**B**). Error bars show standard error of the mean.

electrophoresis assay are not necessarily directly caused by the tested agent, but usually represent intermediate stages in the cellular repair processes (Fortini et al, 1996). This is the case with many alkylating agents, so that the time at which most strand breaks are detected represents a balance between the rate of repair and the rate at which the DNA is initially damaged. In the present study, this point of balance was reached after 2 h exposure, with a return to normal levels of strand breaks after 4 h. However, DNA adducts which might only be detected by further manipulation of the comet assay may still persist in the DNA. It has been suggested that all strand breaks detected by the single-cell gel electrophoresis assay are repaired within 24 h after treatment with a genotoxic agent, but it is by no means certain that no mutagenic events have taken place either before or during the repair processes in a particular region of DNA (Buschfort et al, 1997; Leroy et al, 1996). If the damaged DNA had been processed by an error-prone polymerase that introduced DNA mutations during the repair process, this may in some cases have worse consequences than situations where little or no repair takes place. A cell with highly fragmented DNA is unlikely to proliferate, whereas a cell with uninterrupted DNA strands, even if incorrectly repaired, is likely to multiply and so integrate the mutation into a population of living cells.

The 25 μg spores used in the original extraction procedure corresponds to ca. $10^6$ spores from *P. vittata*, whose spores fall within the middle of the size-range recorded for fern spores. According to current data on outdoor air concentrations of bracken spores, $10^6$ spores is readily inhaled by a person walking for 3 h through areas occupied by sporing bracken (Smith, 1996; Povey et al, 1995). The outcome of the dose-response experiment in the present study suggests that the risk of DNA damage may increase with increasing dose. This is of particular concern to horticultural workers who may be exposed to daily doses in the order of $10^6$ fern spores (Winston, 1998).

Simultaneous incubation with the S9 liver extract completely ablated the strand-breaking effect of the fern spore extracts, indicating that liver enzymes may ameliorate this effect in vivo $P < 0.05$, $n = 4$). This agrees with the findings that bracken-linked cancers are found in the stomach and intestines (see Evans and Mason, 1965), i.e. tissues which the genotoxic agent(s) can affect before being detoxified by the liver. However, bracken is linked to urinary bladder tumours (Bringuier et al, 1995) so that, at least in bracken, there is a component which retains its genotoxic capacity even after passing through the liver.

Spores from one of the tested fern species, *O. regalis*, did not induce DNA damage which was significantly different from the background levels in the various controls (Figure 1). *O. regalis* is the only species of the six tested that produces green spores, which have very thin walls. This may indicate that the DNA-damaging compound(s) are located in the spore wall.

We do not yet know whether in vivo exposure to the tested fern spores is linked to tumourogenesis, but considering the strong mutagenicity and carcinogenicity of the bracken fern, it is reasonable to suppose that it could follow. Furthermore, bearing the relevance of the used method in mind, and considering the strong correlation between DNA damage and carcinogenic events (Fairbairn et al, 1995), we cannot overlook the possibility that the in vitro damage induced by the fern spore extracts have some implications for human health.

### ACKNOWLEDGEMENTS

The authors thank all fern spore collectors who donated the spores used in the experiments, and the FC Moore Studentship Award, the Cancer Research Campaign and the University of Manchester Research Support Fund for financing the study. Many thanks also to an anonymous referee who commented on an earlier draft of the manuscript.

### REFERENCES

Alonso-Amelot ME, Castillo U, Smith BL and Lauren DR (1996) Bracken ptaquiloside in milk. *Nature* **382**: 587

Anderson D, Yu T-W and McGregor DB (1998) Comet assay responses as indicators of carcinogen exposure. *Mutagenesis* **13**: 539–555

Ashby J, Tinwell H, Lefevre PA and Browne MA (1995) The single cell gel electrophoresis assay for induced DNA damage (comet assay) measurement of tail length and moment. *Mutagenesis* **10**: 85–90

 *© 2000 Cancer Research Campaign*

Basaran AA, Yu T-W, Plewa MJ and Anderson D (1996) An investigation of some Turkish herbal medicines in *Salmonella typhimurium* and in the comet assay in human lymphocytes. *Teratog Carcinog Mutagen* **16**: 125–138

Bringuier P-P, Piaton E, Berger N, Debruyne F, Perrin P, Schalken J and Devonec M (1995) Bracken fern-induced bladder tumors in guinea pigs. *Am J Pathol* **147**: 858–868

Bunnag D, Dhorranintra B and Limsuvan S (1989) Ferns and their allergenic importance: skin and nasal provocation tests to fern spore extract in allergic and non-allergic patients. *Annals of Allergy*, **62**: 554–558

Buschfort C, Müller MR, Seeber S, Rajewsky MF and Thomale J (1997) DNA excision repair profiles of normal and leukemic human lymphocytes: functional analysis at the single-cell level. *Cancer Res* **57**: 651–658

Cebulska-Wasilewska A, Nowak D, Niedzwiedz W and Anderson D (1998) Correlations between DNA and cytogenic damage induced after chemical treatment and radiation. *Mutat Res* **421**: 83–91

Conway E (1952) Bracken – the problem plant. *Scottish Agriculture*, Spring: 181–184

Evans IA (1987) Bracken carcinogenicity. In *International quarterly scientific reviews, Reviews on environmental health*, Vol. VII. James GV (ed) pp 161–199. Freund Publishing House Ltd: Tel-Aviv

Evans IA and Mason J (1965) Carcinogenic activity of bracken. *Nature* **208**: 913–914

Fairbairn DW, Olive PL and O'Neill KL (1995) The comet assay: a comprehensive review. *Mutat Res* **339**: 37–59

Fortini P, Raspaglio G, Falchi M and Dogliotti E (1996) Analysis of DNA alkylation damage and repair in mammalian cells by the comet assay. *Mutagenesis* **11**: 169–175

Leroy T, Van Hummelen P, Anard D, Castelain P, Kirch-Volders M, Lauwerys R and Lison D (1996) Evaluation of three methods for the detection of DNA single-strand breaks in human lymphocytes: alkaline elution, nick translation, and single-cell gel electrophoresis. *J Toxicol Environ Health* **47**: 409–422

Ojika M, Wakamatsu K, Niwa H and Yamada K (1987) Ptaquiloside, a potent carcinogen isolated from bracken fern *Pteridium aquilinum* var. *latiusculum*: structure elucidation based on chemical and spectral evidence and reactions with amino acids, nucleosides and nucleotides. *Tetrahedron* **43**: 5261–5274

Östling O and Johanson K (1984) Microelectrophoretic study of radiation-induced DNA damages in individual mammalian cells. *Biochem Biophys Res Commun* **123**: 291–298

Page CN (1979) Experimental aspects of fern ecology. In *The experimental biology of ferns*. Dyer AF (ed.) pp 105–140. Academic Press: London

Pamukcu AM (1963) Epidemiologic studies on urinary bladder tumors in Turkish cattle. *Ann N Y Acad Sci* **108**: 938–947

Povey AC, Evans IA, Taylor JA and O'Connor PJ (1995) Detection of DNA adducts by $^{32}$P-postlabelling in mice treated with bracken extract and bracken spores. In *Bracken: an environmental issue*. Smith RT and Taylor JA (eds) pp 95–98. International Bracken Group: Aberystwyth

Povey AC, Evans IA, Taylor JA and O'Connor PJ (1996) $^{32}$P-post-labelling analysis of DNA adducts formed in the upper gastrointestinal tissue of mice fed bracken extract or bracken spores. *Br J Cancer* **74**: 1342–1348

Prakash AS, Pereira TN, Smith BL, Shaw G and Seawright AA (1996) Mechanism of bracken fern carcinogenesis: evidence for H-*ras* activation via initial adenine alkylation by ptaquiloside. *Natural Toxins* **4**: 211–227

Simán SE and Sheffield E (2000) *Polypodium vulgare* plants transferred from their natural habitat to a glasshouse sporulate continuously. *American Fern Journal* (accepted)

Simán SE, Povey AC and Sheffield E (1999) Human health risks from fern spores? A review. *Fern Gazette* **15**: 275–287

Smith M (1996) Spores for thought. *Independent on Sunday* **Sep 22**: 51

Villalobos-Salazar J, Meneses A and Salas J (1990) Carcinogenic effects in mice of milk from cows fed bracken fern *Pteridium aquilinum*. In *Bracken biology and management*. Thompson JA and Smith RT (eds) pp 247–251. Australian Institute of Agricultural Science, Occasional publication No. 40: Australia

Villalobos-Salazar J, Mora J, Meneses A and Pashov B (1995) The carcinogenic effects of bracken spores. In *Bracken: an environmental issue*. Smith RT and Taylor JA (eds) pp 102–103. International Bracken Group: Aberystwyth

Ward TH, Butler J, Shahbakhti H and Richards JT (1997) Comet assay studies on the activation of two diaziridinylbenzoquinones in K562 Cells. *Biochem Pharmacol* **53**: 1115–1121

Wagner ED, Rayburn AL, Anderson D and Plewa MJ (1998) Analysis of mutagens with single cell gel electrophoresis, flow cytometry, and forward mutation assays in an isolated clone of Chinese hamster ovary cells. *Environ Mol Mutagen* **32**: 360–368

Winston D (1998) *Risk assessment of environmental exposure to fern spores* MSc Thesis, The University of Manchester

*© 2000 Cancer Research Campaign*
